# Morvan’s syndrome with hypercoagulable condition in a patient positive for anti-CASPR2 antibodies: A case report

**DOI:** 10.1097/MD.0000000000038929

**Published:** 2024-07-19

**Authors:** Xintong Pang, Yanming Li, Zining Liu, Yafei Mao, Xinyuan Li, Lingling Gao, Yulan Geng, Li Meng

**Affiliations:** a Department of Laboratory Medicine, The First Hospital of Hebei Medical University, Shijiazhuang, China; b Department of Neuroimmunology, The First Hospital of Hebei Medical University, Shijiazhuang, China.

**Keywords:** anti-CASPR2 antibody, hypercoagulable state, Morvan’s syndrome, neuroinflammation, procoagulant factors

## Abstract

**Rationale:**

The phenomenon of hypercoagulability has not been previously documented in individuals with Morvan’s syndrome, especially in those associated with contactin-associated protein-like receptor 2 (CASPR2).

**Patient concerns:**

A previously healthy 32-year-old Chinese male was admitted to the hospital with central and peripheral neurologic symptoms. The patient was tested positive for anti-CASPR2 antibodies, and also presented with an activated coagulation state on admission, characterized by a low activated partial thromboplastin time and a high platelet count. With gradual improvement of clinical symptoms, activated partial thromboplastin time, and platelet count returned to normal. Simultaneously, anti-CASPR2 antibody titers significantly decreased and eventually became undetectable.

**Diagnoses:**

The patient was diagnosed as Morvan’s syndrome with positive anti-CASPAR2 antibodies accompanied with hypercoagulable state.

**Interventions:**

Plasmapheresis was administered to improve the symptoms combined with prednisolone acetate therapy.

**Outcomes:**

The patient experienced complete resolution of all symptoms during hospitalization and generally recovery after 2 months of discharge.

**Lessons:**

Emphasis should be directed towards hypercoagulability in individuals diagnosed with Morvan’s syndrome, particularly those presenting with positive anti-CASPR2 antibodies. Anticoagulant therapy may represent a novel therapeutic approach for individuals afflicted with Morvan’s syndrome and exhibiting positivity for anti-CASPR2 antibodies.

## 1. Introduction

A preexisting hypercoagulable state is a risk factor for the development of venous thromboembolism. The primary cause of acquired hypercoagulability is an increase in procoagulant factors and/or a decrease in natural inhibitors. Surgery, pregnancy, contraceptive use, cancer, and traveler’s thrombosis (economy class syndrome) represent prominent high-risk conditions that pose significant challenges in inducing hypercoagulability.^[1]^ In a study, it was indicated that hypercoagulability may exacerbate insulin resistance, neuroinflammation, blood‐brain barrier disruption, and amyloid β-protein accumulation in neurodegenerative diseases, such as Alzheimer’s disease.^[2]^ However, there is no published literature on Morvan’s syndrome with hypercoagulability or thrombosis. Here, a case of Morvan’s syndrome accompanied with hypercoagulable state is reported. The syndrome is associated with positive anti-contactin-associated protein-like receptor 2 (CASPR2).

## 2. Case description

On June 9, 2023, a 32-year-old male of Chinese ethnicity, employed as a deliveryman, was admitted to the psychiatric department of The First Hospital of Hebei Medical University (refer Table 1). The patient reported no prior history of medication. Notably, 2 months preceding admission, the patient experienced symptoms of nausea and vomiting subsequent to alcohol consumption. Subsequent to the initial onset, the patient exhibited a progression of pain extending from the lower limbs to the upper limbs. Seeking relief from the persistent and exacerbating pain in the limbs, the patient sought consultation at various medical facilities. Regrettably, concomitant with these symptoms, the patient reported episodes of “muscle twitching” in the legs, as visually documented in OA_Supplemental Digital Content, http://links.lww.com/MD/N220. Concomitantly, the individual presented with mood swings, severe insomnia, short-term memory dysfunction, and a notable weight loss of 20 kg. Despite undergoing anti-anxiety treatment, there was no discernible improvement in the patient’s condition.

**Table 1:** The treatment process of the patient.

Time	The treatment process
May 6, 2023	The Chinese man showed nausea and vomiting after a drink.
During May 6, 2023 and June 8, 2023	He visited 5 different hospitals for gastrointestinal dysfunction, pain of his limbs, mood fluctuation, severe insomnia, short-term memory dysfunction, and a 20 kg weight loss.
June 9, 2023	The Chinese man presented to the Department of Psychiatry of our hospital.
June 15, 2023	The patient was transferred to the Department of Neuroimmunology of our hospital.
June 16, 2023	Morvan’s syndrome with positive anti-CASPR2 was demonstrated, and the prednisolone acetate was scheduled.
June 23, 2023 June 24, 2023	The first plasmapheresis was performed. The blood clot was found in the mixer of plasma exchange instrument. The second plasmapheresis was performed.
June 26, 2023	The third plasmapheresis ended and the patient’s manifestations were markedly alleviated.
June 29, 2023	The patient’s clinical features and clinical makers returned to generally normal. He was discharged with the advice of prednisolone acetate and more attention on hypercoagulability.

On admission, the patient exhibited severe insomnia, mood swings, cognitive impairment, hallucinations, and other forms of mental disorders. The initial clinical examination revealed a body temperature of 36.0°C, a heart rate of 120 beats/minute, a respiratory rate of 18 beats/minute, and blood pressure of 108/81 mm Hg. The adverse event score was 8 points, with 3 points attributed to mental disorder, 1 point to language, 1 point for walking, and 1 point for muscle strength. The electrocardiogram showed QTc prolongation of 495 ms (generally <440 ms). Imaging of the head, lungs, heart, abdomen, and pelvis revealed no abnormalities. Routine blood tests revealed abnormal lymphocyte percentage, red blood cell count, packed cell volume, neutrophil count, monocyte count, and platelet count (refer Table 2). Routine urine and stool tests were negative. The biochemical examination revealed lower levels of serum potassium (3.39 mmol/L) (reference range: 3.50–5.30 mmol/L), total protein (57.6 g/L) (reference range: 65.0–85.0 g/L), and albumin (38.0 g/L) (reference range: 40.0–55.0 g/L). Coagulation test revealed approximately normal prothrombin time, activated partial thromboplastin time (APTT), thrombin time, fibrinogen, D-dimer, plasminogen activity, fibrin/fibrinogen degradation products, and antithrombin III activity (Table 3). Thyroid function test revealed that total triiodothyronine decreased by 0.66 nmol/L (reference range: 1.02–2.48 nmol/L) and free thyroxine increased by 24.04 pmol/L (reference range: 7.60–16.10 pmol/L). Serum hormone examination revealed that testosterone decreased by 1.66 ng/mL (reference range: 1.75–7.81 ng/mL) and prolactin increased by 19.57 ng/mL (2.64–13.13 ng/mL). Examinations for hepatitis B virus, hepatitis C virus, HIV, *Treponema pallidum*, and other infectious pathogens revealed no discernible abnormalities. Cerebrospinal fluid (CSF) analysis revealed elevated protein of 1180.4 mg/L (reference range: 0–150 mg/L). On June 15, 2023, the patient was transferred to the neuroimmunology department due to the manifestation of a biological mental disorder.

**Table 2:** Blood routine changes in the course of hospitalization.

Project	June 9, 2023	June 11, 2023	June 14, 2023	June 16, 2023	June 21, 2023	June 24, 2023	August 4, 2023	Reference range
White blood cell count	9.3	11.3	16.8	8.1	8.4	8.6	8.6	3.5–9.5 × 10^9^/L
Neutrophil percentage	74.2	74.6	88.8	70.6	89.4	77.0	74.8	40.0–75.0%
Neutrophil count	6.90	8.40	14.90	5.71	7.50	6.60	6.43	1.80–6.30 × 10^9^/L
Lymphocyte percentage	17.2	18.0	7.1	18.8	8.3	15.2	17.9	20.0–50.0%
Lymphocyte number	1.60	2.00	1.20	1.53	0.70	1.30	1.54	1.10–3.20 × 10^9^/L
Red blood cell count	3.91	4.38	4.62	4.36	4.07	3.88	4.24	4.30–5.80 × 10^12^/L
Hemoglobin	130	147	155	145	133	126	140	130–175 g/L
Hemocrit	36.7	41.8	44.0	41.6	40.0	37.9	40.5	40.0–50.0
Platelet count	488	491	472	359	361	247	251	125–350 × 10^9^/L

**Table 3:** Alterations in coagulation markers during hospitalization.

Project	June 9, 2023	June 16, 2023	June 24, 2023	July 18, 2023	August 4, 2023	Reference range
PT	11.2	10.7	11.3	11.1	12.0	9.4–12.5 s
APTT	25.9	25.0	23.8	26.5	27.2	25.1–36.5 s
TT	15.6	12.0	15.3	12.7	13.6	10.3–16.6 s
Fibrinogen	2.90	4.28	2.49	2.87	2.23	2.38–4.98
D-dimer	0.43	0.25	0.46			0–0.55 mg/L
FDP		0.74	0.90			0–5.00 mg/L
FLG-A		82.0	83.0			80–130%
Antithrombin III		121	114			83–128%

APTT = activated partial thromboplastin time, FDP = fibrin/fibrinogen degradation product, PLG-A = plasminogen activity, PT = prothrombin time, TT = thrombin time.

The patient underwent other tests in the neuroimmunology department. The neurological examination revealed that the patient had peripheral nervous system damage (such as muscular dystrophy in hands and limbs, muscle weakness, numbness in hands and feet, and pain in the limbs) and central nervous system damage (such as positive Babinski reflex of the right foot). Electromyography revealed slow motor and sensory nerve conduction velocities. EEG showed a significant increase in signal power in the θ wave. Immuno-inflammatory testing revealed abnormalities in complements (Cs), immunoglobulins (Igs), cytokines, C-reactive protein, and lymphocyte subsets (Table 4). The serum carcinoembryonic antigen level was 17.93 ng/mL (reference range: 0–5.00 ng/mL). The serum homocysteine concentration was 16.29 μmol/L (reference range: 0–15.00 μmol/L).

**Table 4:** Immunoinflammation-related markers on admission.

Project	Result			Reference range	Project	Result	Reference range	Project	Results	Reference range	
Lymphocyte subsets					Inflammatory panel			Complements-immunoglobulins panel			
T%		71.68	64.60–77.10%		IL-1	0.12	0–12.40 pg/mL	C1q	160		159–233mg/L
CD4 + T%		38.40	32.70–44.20%		IL-2	0.79	0–5.71 pg/mL	C3	0.82		0.90–1.80 g/L
CD8 + T%		28.96	24.80–36.00%		IL-4	1.78	0–3.00 pg/mL	C4	0.20		0.10–0.40 g/L
CD4+/CD8+		1.33	0.80–1.72		IL-5	3.97	0–3.10 pg/mL	BF-1	285.6		100.0–400.0 mg/L
B%		12.99	14.50–30.30%		IL-6	8.78	0–7.00 pg/mL	IgA	1.67		0.70–4.00 g/L
NK%		11.98	6.40–12.50%		IL-8	35.44	0–20.60 pg/mL	IgG	6.96		7.00–16.00 g/L
L count		1530	1100–3200 cells/μL		IL-10	0.56	0–4.91 pg/mL	IgM	0.84		0.40–2.30 g/L
T count		1097	955–2860 cells/μL		IL-17A	1.57	0–20.60 pg/mL	IgE	140		0–375 IU/mL
CD4 + T count		588	550–1440 cells/μL		IL-12P70	0.12	0–3.40 pg/mL				
CD8 + T count		443	320–1250 cells/μL		IFN-α	4.26	0–8.50 g/mL				
B count		199	90–360 cells/μL		IFN-γ	3.21	0–7.42 pg/mL				
NK count		183	150–1100 cells/μL		TNF-α	0.12	0–4.60 pg/mL				

B = B lymphocyte, BF = B factor, C = complement, CRP = C-reactive protein, IFN = interferon, Ig = immunoglobulin, IL = interleukin, L = lymphocyte, NK = nature killer, T = T lymphocyte, TNF = tumor necrosis factor.

**Figure 1.**
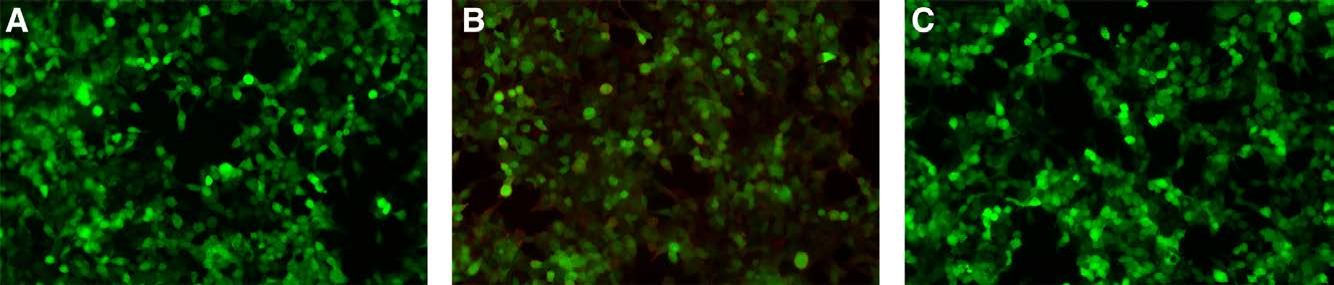
The immunoreactivity of the CSF and serum in the patient to CASPR2 proteins with indirect immunofluorescence test. (1A) The patient’s CSF collected on June 13, 2023 showed moderate binding to the surface of cells expressing CASPR2 proteins (1:1 dilution). (1B) The patient’s serum collected on June 17 showed weak binding to the surface of cells expressing CASPR2 proteins (1:100 dilution). (1C) The patient’s serum collected on June 27 showed moderate binding to the surface of cells expressing CASPR2 proteins (1:10 dilution). CSF = cerebrospinal fluid and CASPR2 = contactin-associated protein-like receptor 2.

A comprehensive assessment of serum and CSF was conducted to diagnose autoimmune diseases and paraneoplastic encephalitis. Notably, positive CASPR2 antibodies were identified, with a dilution of 1:100 in serum and 1:1 in CSF, as illustrated in Figure 1A–C.

Based on clinical and laboratory results, the patient received a diagnosis of Morvan’s syndrome.^[3]^ Subsequently, the prescribed treatment regimen included prednisone acetate, supplemented by a three-day course of psychotherapeutic intervention with risperidone. Unfortunately, there was no significant relief in the symptoms. Plasma exchange was arranged for the patient as per the recommendations of the German Autoimmune Encephalitis Research Network (https://en.generate-net.de/how-do-we-treat-autoimmune-encephalitis.html) and the Neurological Syndrome Treatment Guidelines by the American Society for Apheresis (ASFA).^[4]^ Following three cycles of plasma exchange, the patient exhibited significant improvements in insomnia, limb pain, mood disorders, and other neurological dysfunctions.

**Figure 2.**
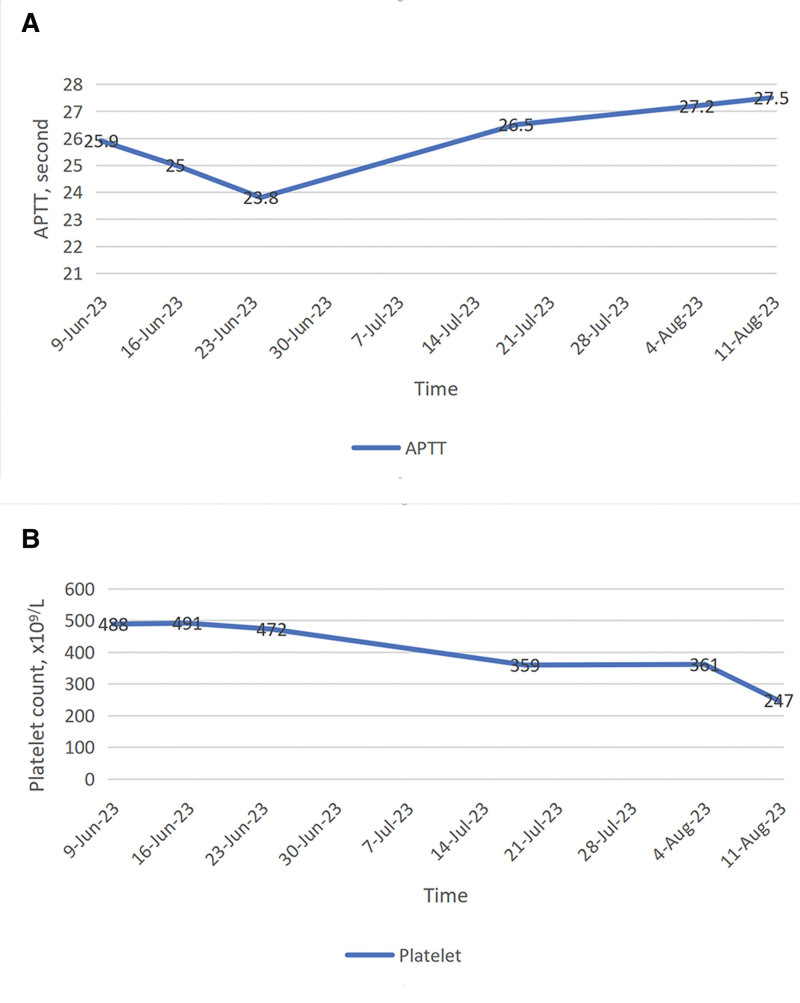
The variations in both APTT and platelet count during hospitalization. (2A) Variations in APTT; APTT was from low to normal during recovery. (2B) Variations in platelet count. Platelet count was from high to normal during recovery. APTT = activated partial thromboplastin time.

It is worth noting that during the initial cycle of plasma exchange, a blood clot was discovered at the point where the anticoagulant and blood were mixed in a ratio of 1:12 at the intersection of the plasma exchange machine. After changing the mixing ratio to 1:11, no abnormalities were detected subsequently. Laboratory data revealed that plasma APTT was low and platelet count was high on admission. As the patient recovered, these abnormal indicators causing the hypercoagulable state gradually returned to an approximately normal state (Fig. 2A and B). All of the above suggests that the patient suffered from Morvan’s syndrome and a hypercoagulable state. Within a span of 3 days, the patient experienced complete resolution of all symptoms; however, only partial relief was observed in terms of myasthenia. The patient was discharged and continued to take prednisone acetate. Concurrently, rigorous monitoring of coagulation indicators was undertaken, while regular assessments of head MRI, CASPR2 antibodies, APTT, platelet count, and other pertinent laboratory markers were conducted. At the 2-month follow-up visit, muscle weakness improved significantly, and the patient was able to walk independently.

All procedures performed in this study were in accordance with the ethical standards of the institutional and/or national research committee and as per the Declaration of Helsinki (2013 revision). Written informed consent for publication was obtained from the patient.

## 3. Discussion

This case presents Morvan’s syndrome associated with aberrations in the CASPR2 protein, concomitant with a concurrent hypercoagulable state. Morvan’s syndrome is an inflammatory disease of the nervous system caused by 1 or several specific autoimmune antibodies that may lead to brain and peripheral neuropathy by disrupting axonal potassium currents. Abnormal immune mechanisms play a crucial role in the pathogenesis of Morvan’s syndrome. Generally, B lymphocytes differentiate in to plasma cells to produce antibodies that exert their effects through C-dependent or C-independent mechanisms (such as anti-CASPR2 IgG targets multiple epitopes of the CASPR2 protein).^[5,6]^ Various T lymphocytes (CD4 + T and CD8 + T) and natural killer cells help destroy/kill target cells. In such a case, a decrease in the percentage of B lymphocytes, IgG, C1q, and C3, as well as counts of effector cells such as CD3 + T, CD8 + T, and natural killer cells near the lower limit of the reference range indicate depletion of immune cells and immune molecules. This depletion is a clear sign of immune activation. A study on anti-NMDAR encephalitis, which included 19 articles and a total of 579 patients, revealed that multiple immune cells, including T cells and B cells, play an important role in the pathology.^[7]^ It was also discovered that the functional manifestation of immune cells included cytokine/chemokine profiles such as increased interleukin (IL) IL-6, tumor necrosis factor-α, IL-10, IL-1β, IL-12, IL-17, and IL-2 in CSF. The patient in this case had elevated serum cytokine/chemokine levels, such as IL-5, IL-6, and IL-8. This variation may be due to disparities in populations, encephalitis-related antibodies, sample types, and sampling intervals.

Two studies have documented the presence of hypercoagulable state in NMDA-related or GABA-related autoimmune encephalitis.^[8,9]^ Morvan’s syndrome represents another variant within the spectrum of autoimmune encephalitis. It is noteworthy to highlight that the patient described in this case of Morvan’s syndrome also presented with a hypercoagulable state, as evidenced by a low APTT and an elevated platelet count. In this case, the hypercoagulable state may be related to the following factors. First, the patient had a high platelet count. High concentrations of platelets contribute to hemostasis and neuroinflammation.^[10]^ The thrombotic response is crucial In the inflammatory process. Platelets expressing toll-like receptors (TLRs) 2 may also accelerate thrombosis or coagulation activation.^[11]^ Second, abnormalities in C levels, including C1q and C3, were observed in the patient. An imbalance between C activation and regulation at the host surface is implicated in diseases predispose individuals to severe thrombotic events.^[12]^ Third, the patient had lower lymphocyte subpopulations. Increased neutrophil counts and elevated fibrinogen levels indirectly enhance venous thrombosis in mice with B cell deficiency.^[13]^ In contrast, T-cell activation promotes platelet aggregation through increased cytotoxic T cells (CD8 + T) and T helper cells (CD4 + T) mediated by platelet GPIIb/IIIa, CD40L, and the lymphocyte integrin αM.^[14]^ Decreased B-cell and T-lymphocyte subsets contributed to the hypercoagulable state of the patient. Fourth, the patient exhibited elevated levels of IL-8 and neutrophile granulocytes, which were characterized by the formation of degranulated and neutrophil extranuclear traps (NETs). Such neutrophils activate coagulation and C cascade reactions. A marked immunothrombotic state has been observed in patients with infections.^[15]^ Fifth, the serum prolactin level in this patient was found to be abnormal. By blocking dopamine D2 receptors, antipsychotic drugs inhibit the sustained release of dopamine in the pituitary gland and enhance prolactin secretion.^[16]^ Patients with prolactinoma have significantly increased platelet counts, fibrinogen, and antithrombin III, and decreased plasma tissue factor pathway inhibitors.^[17]^ Prolactin is also a potent co-activator of platelet aggregation. High-density prolactin can enhance ADP-stimulated platelet aggregation through Gq protein, thereby inducing alterations in the shape of activated platelets.^[16]^ All these factors led to a hypercoagulable state in the patient.

In conclusion, the hypercoagulable state observed in Morvan’s syndrome is multifaceted. Factors including platelets, prolactin, and immune-inflammatory elements may contribute to the complexity of the hypercoagulable state in Morvan’s syndrome. These findings suggest that anticoagulant therapy could represent a novel therapeutic avenue for addressing Morvan’s syndrome, particularly in individuals identified with positive anti-CASPR-2 antibodies.

## Author contributions

**Conceptualization:** Xintong Pang, Yafei Mao, Lingling Gao, Yulan Geng.

**Data curation:** Xintong Pang, Yanming Li, Zining Liu, Xinyuan Li, Yulan Geng.

**Formal analysis:** Zining Liu, Yafei Mao, Xinyuan Li, Lingling Gao.

**Methodology:** Yanming Li, Li Meng.

**Project administration:** Li Meng.

**Visualization:** Yafei Mao.

**Writing ‐ original draft:** Xintong Pang, Yulan Geng, Li Meng.

**Writing ‐ review & editing:** Yanming Li, Zining Liu, Xinyuan Li, Lingling Gao, Yulan Geng.

## Supplementary Material


